# Auditory Stimulation-Evoked Frontoparietal Theta Asymmetry for Subtype Classification and Long-Term Prognosis Prediction in Disorders of Consciousness

**DOI:** 10.3390/brainsci16101138

**Published:** 2026-10-21

**Authors:** Chen Chen, Yajuan Hu, Sifan Wang, Xintong Zhang, Shuang Zheng, Jinying Han, Kai Wang

**Affiliations:** 1 Department of Neurology, The First Affiliated Hospital of Anhui Medical University, Hefei 230032, China; yfy1262705@fy.ahmu.edu.cn (C.C.); yfy103955@fy.ahmu.edu.cn (Y.H.); sf11042023@163.com (S.W.); zxt113@126.com (X.Z.); m15212785951_1@163.com (S.Z.); 2346010132@stu.ahmu.edu.cn (J.H.); 2 Anhui Province Key Laboratory of Cognition and Neuropsychiatric Disorders, Hefei 230032, China; 3 Collaborative Innovation Centre of Neuropsychiatric Disorder and Mental Health, Hefei 230032, China; 4 The School of Mental Health and Psychological Sciences, Anhui Medical University, Hefei 230032, China; 5 Institute of Artificial Intelligence, Hefei Comprehensive National Science Center, Hefei 230032, China; 6 Anhui Institute of Translational Medicine, Hefei 230032, China

**Keywords:** DOC, MCS, VS, asymmetry, auditory stimulation, prognosis

## Abstract

**Highlights:**

**What are the main findings?**

- Auditory stimulation evokes asymmetric frontoparietal theta oscillations that distinguish disorders of consciousness subtypes: vegetative state (VS) vs. minimally conscious state (MCS).
- Frontoparietal theta asymmetry predicts long-term functional outcome beyond conventional behavioral assessment.

**What are the implications of the main findings?**

- Theta asymmetry offers an objective electrophysiological marker for subtype classification in non-communicative patients.
- This biomarker may facilitate early prognostication and individualized rehabilitation planning in disorders of consciousness.

**Abstract:**

**Background**: Quantitative electroencephalography (qEEG) facilitates the objective assessment of residual brain function in patients with disorders of consciousness (DOC), which addresses the intrinsic limitations of behavioral assessment methods. Nevertheless, the characteristics of frontoparietal neural oscillation asymmetry between resting state and auditory stimulation state have not been fully elucidated, and its incremental prognostic value for DOC subtype classification and outcome prediction remains to be confirmed. **Methods**: Fifty-seven patients with DOC and 23 healthy controls completed qEEG recordings at baseline and during auditory stimulation. Interhemispheric asymmetry indices of the frontal lobe and parietal lobe were calculated for the theta band and alpha band. Linear mixed-effects models (LMMs) were adopted to test the main effects and interaction effects of diagnosis, brain region and stimulation condition. The correlations between asymmetry indicators, Coma Recovery Scale-Revised (CRS-R) scores and 6-month Glasgow Outcome Scale (GOS) scores were analyzed. Nested logistic regression and Receiver Operating Characteristic (ROC) analysis were performed to quantify the incremental prognostic value of oscillatory asymmetry beyond conventional clinical variables; odds ratios (ORs) with 95% confidence intervals (CIs) were calculated for all predictive indicators. Internal leave-one-out cross-validation and external cohort validation were performed to assess the generalizability and robustness of the final prognostic model. **Results**: Significant diagnosis × region interaction effects were observed in both frequency bands. Vegetative state (VS) patients presented with frontal-dominant asymmetry, while minimally conscious state (MCS) patients showed parietal-dominant asymmetry; this dissociative pattern was significantly amplified after auditory stimulation. Only stimulation-evoked theta asymmetry showed consistent correlations with clinical status and prognosis: frontal theta asymmetry was negatively correlated with CRS-R scores, while parietal theta asymmetry was positively correlated with CRS-R scores and 6-month GOS outcomes. Incorporating stimulated frontoparietal theta asymmetry significantly improved prognostic performance, and the area under the curve (AUC) of the optimal model reached 0.930. **Conclusions**: VS and MCS present distinct frontoparietal oscillatory asymmetry phenotypes, which are specifically enhanced by auditory stimulation. Stimulation-evoked theta band asymmetry can reflect residual conscious function and provides incremental prognostic value for long-term functional recovery, with robust generalizability verified by internal and external validation.

## 1. Introduction

Severe acquired brain injury frequently induces disorders of consciousness (DOC), which poses substantial clinical challenges and imposes considerable socioeconomic burdens on a global scale [1]. According to standardized behavioral criteria, DOC is primarily categorized into the vegetative state (VS), defined by a complete absence of subjective conscious awareness, and the minimally conscious state (MCS), characterized by preserved partial conscious processing capacity [2,3]. Accurate subtype differentiation and early long-term prognostication remain unmet critical clinical needs in DOC management. Bedside behavioral assessments based on the Coma Recovery Scale-Revised (CRS-R) are constrained by inherent limitations, including fluctuating arousal levels, patient fatigue, and covert residual consciousness, which frequently lead to diagnostic misclassification and inaccurate prognostic judgment [1]. In this context, objective quantitative neurophysiological biomarkers are urgently needed to complement conventional behavioral evaluation systems and optimize clinical decision-making.

Prevailing theoretical frameworks underscore the pivotal role of the frontoparietal network in sustaining consciousness. The Global Workspace Theory posits this network as the central hub for global information broadcasting, whereas the Integrated Information Theory emphasizes posterior parietal cortical integration [4]. Although these models motivate the selection of frontal and parietal regions as critical nodes, they do not inherently predict interhemispheric asymmetry. Rather, the hypothesis that left–right frontoparietal oscillatory imbalance differentiates VS from MCS is grounded in convergent empirical evidence: prior EEG and neuroimaging studies have documented disease-specific hemispheric disconnection and interhemispheric imbalance in DOC [5,6], and stroke cohorts have validated oscillatory asymmetry as a robust biomarker for neurological dysfunction and recovery [7,8]. Accordingly, we focused on homologous frontal and parietal electrode pairs to test whether interhemispheric oscillatory asymmetry within these core conscious hubs can distinguish DOC subtypes and predict functional outcome.

EEG has emerged as a reliable, bedside-accessible modality for evaluating residual thalamo-cortical and frontoparietal network function in DOC patients. Cumulative evidence demonstrates that alpha- and theta-band spectral power, functional connectivity, and oscillatory dynamics are closely correlated with the degree of residual conscious function [9,10,11,12]. The frontoparietal neuronal workspace constitutes the core substrate for conscious information integration, and network disruption within this system serves as a fundamental pathophysiological distinction between VS and MCS [13,14]. To date, cortical asymmetry research has predominantly focused on psychiatric disorders, including depression and schizophrenia [15,16,17], as well as neurodegenerative diseases such as Alzheimer’s disease [18]. In stroke cohorts, oscillatory asymmetry has been validated as a robust biomarker for neurological dysfunction and short-term functional recovery [7,8]. However, existing DOC quantitative electroencephalography (qEEG) studies have critical gaps: nearly all prior asymmetry analyses only adopted resting-state recordings with single frequency bands, none systematically compared resting and auditory-stimulated oscillatory asymmetry across both alpha and theta bands, and no prior work quantified the independent incremental prognostic value of state-specific frontoparietal asymmetry via nested multivariable regression models. Furthermore, no prior investigations have clarified the frequency-specific clinical relevance of such asymmetry or its incremental prognostic value for long-term functional recovery in DOC populations.

Against this backdrop, the present study addressed three hierarchical research objectives: (1) to determine whether patients with VS and MCS exhibit distinct frontoparietal interhemispheric oscillatory asymmetry patterns at rest and following auditory stimulation; (2) to clarify whether alpha- and theta-band asymmetry indices display differential associations with consciousness assessment and long-term functional outcomes; and (3) to verify whether the integration of stimulation-evoked frontoparietal asymmetry can improve early prognostic performance for 6-month functional recovery in DOC patients.

We hypothesized that VS and MCS possess unique subtype-specific frontoparietal oscillatory asymmetry phenotypes that can be dynamically amplified by auditory stimulation, and that stimulation-modulated frequency-specific asymmetry serves as an optimized neurophysiological biomarker for precise DOC subtyping and reliable long-term prognostication.

## 2. Methods

### 2.1. Participants

This study recruited 57 patients with DOC and 23 healthy control participants from the First Affiliated Hospital of Anhui Medical University. Eligibility criteria were as follows: (1) A diagnosis of DOC confirmed via the CRS-R assessment performed within 72 h prior to EEG recording; (2) age ≥ 18 years; (3) cranial computed tomography (CT) or magnetic resonance imaging (MRI) verifying intracranial lesions as the primary etiology of DOC; (4) no sedative agents administered within 24 h before EEG acquisition; (5) no premorbid hearing impairment or peripheral auditory pathway lesions, confirmed via review of pre-injury medical records, caregiver interviews, and bedside neurological and otological examinations; (6) written informed consent obtained from all healthy volunteers and legal surrogates of patients with DOC. Participants were excluded if they satisfied any of the following criteria: (1) pre-existing severe neurological disorders unrelated to the index brain injury responsible for DOC; (2) contraindications to EEG recording, including extensive skull defects or persistent severe agitation that precluded satisfactory data acquisition; (3) unstable vital signs hindering complete implementation of the experimental paradigm. All participants received standard clinical care during the study period. This study was reviewed and approved by the Ethics Committee of the First Affiliated Hospital of Anhui Medical University (Approval No. PJ2020-17-11) and conducted in compliance with relevant clinical research regulations and guidelines.

For external prognostic validation, a total of 37 independent DOC inpatients recruited from the same hospital during a subsequent enrollment period were included. All external patients followed identical inclusion and exclusion criteria, standardized qEEG acquisition paradigms, baseline CRS-R assessments, and 6-month Glasgow Outcome Scale (GOS) outcome collection procedures as the primary derivation cohort.

### 2.2. Behavioral Assessment and Prognostic Classification

All patients underwent standardized behavioral consciousness evaluation using the CRS-R, performed independently by two experienced attending physicians prior to EEG acquisition. Each patient received a minimum of five repeated CRS-R assessments within one week before enrollment, with the highest recorded score adopted for final subtype classification. As a validated gold-standard scale for DOC evaluation, the CRS-R assesses six functional subdomains covering auditory, visual, motor, verbal, communication, and arousal functions, with total scores ranging from 0 to 23, where higher scores reflect better-preserved conscious function [19]. All participants completed structured telephone follow-up at six months post-enrollment, with long-term functional outcomes evaluated using GOS. Prognostic endpoints were dichotomized for statistical analysis: a GOS score ≥ 3 was defined as a favorable functional outcome, whereas scores of 1–2 indicated unfavorable persistent neurological impairment [20].

### 2.3. Auditory Stimuli

A clipped segment of the traditional Chinese folk melody *Moon Reflected on the Second Spring* was utilized as the auditory stimulus. This piece was selected for its high cultural familiarity and emotional salience in Chinese populations, which can engage limbic and autobiographical memory networks to a greater extent than neutral tones. All patients wore noise-canceling headphones to ensure isolated and uniform auditory input. Audio stimuli were delivered via computer at a calibrated constant volume of 75 dB. Prior to stimulation, resting-state EEG signals were continuously recorded for at least 8 min to establish stable baseline data. During auditory stimulation, EEG signals were synchronously and continuously acquired for subsequent state-dependent comparative analysis.

### 2.4. EEG Data Acquisition and Pre-Processing

Continuous EEG signals were recorded for a minimum of 8 min using a standard 19-channel Ag/AgCl pin-type scalp electrode montage. All electrodes were connected to a polygraphic amplifier (EEG-1200C; Nihon Kohden, Tokyo, Japan) with a sampling rate of 200 Hz. Skin-electrode impedance was maintained below 5 kΩ throughout recording to guarantee high signal quality. Offline preprocessing was conducted via EEGLAB v13.0b within MATLAB v2013b (MathWorks Inc., Natick, MA, USA). Raw EEG data were first processed with a 50 Hz notch filter to eliminate power-line artifacts, followed by bandpass filtering between 0.1 Hz and 40 Hz [21]. Independent component analysis (ICA) was subsequently applied to identify and remove ocular and muscular artifacts [22]. Clean EEG epochs were re-referenced to the common average reference, and segments with peak-to-trough amplitudes exceeding ±150 μV were excluded to ensure data reliability. The bedside recording setup is illustrated in Supplementary Figure S1.

### 2.5. Calculation of Cerebral Asymmetry

Artifact-free EEG epochs were processed via fast Fourier transformation (FFT) to extract spectral power within theta (4–8 Hz) and alpha (8–13 Hz) frequency bands. Absolute spectral power (μV^2^) was calculated for homologous frontal (F3/F4) and parietal (P3/P4) electrode pairs to quantify region-specific interhemispheric asymmetry [23]. Frontal and parietal laterality quotient (LQ) indices were computed using logarithmic transformation to eliminate systematic frequency-band power differences and generate normally distributed asymmetry values. LQ was defined as LQ = ln(right power) − ln(left power), yielding F0 (frontal LQ at baseline), F1 (frontal LQ under auditory stimulation), P0 (parietal LQ at baseline), P1 (parietal LQ under auditory stimulation) for subsequent statistical comparison [24].

### 2.6. Statistical Analysis

All statistical analyses were performed using SPSS 26.0 and R software (v4.6.1), with a two-tailed α = 0.05 significance threshold. The Shapiro–Wilk test was used to evaluate data normality. Normally distributed variables were presented as mean ± standard deviation, while non-normal data were reported as median (25th, 75th percentile). Intergroup demographic and behavioral differences were compared using independent t-tests or Mann–Whitney U tests accordingly. The Benjamini–Hochberg false discovery rate correction was applied for all multiple comparisons to control Type I error inflation.

Linear mixed-effects models (LMMs) with random participant intercepts were constructed to assess dynamic alterations in frontoparietal oscillatory asymmetry across diagnostic subgroups, brain regions, and stimulation conditions, with age and sex included as confounding covariates. Sensitivity analyses incorporating additional clinical covariates (DOC duration, lesion location, and injury etiology) confirmed that the core Group × Region interaction effect remained statistically significant. Separate models were fitted for alpha and theta bands, with auditory stimulation status, brain region, DOC subtype, and their three-way interaction defined as fixed effects. All models were implemented via the lmerTest package using restricted maximum likelihood estimation. Type III fixed-effect tests with Satterthwaite approximation were applied to calculate denominator degrees of freedom. Upon detection of significant interaction effects, stratified post hoc pairwise comparisons were performed using the emmeans function, with the Kenward–Roger method used for degrees of freedom correction and Bonferroni adjustment applied to control familywise error rates within each contrast set. Pairwise comparisons systematically evaluated subtype differences across conditions, state changes between baseline and stimulation within each subgroup, and frontoparietal regional differences across diagnostic and experimental states.

Spearman’s rank correlation analyses were performed to examine associations between frontoparietal asymmetry indices and clinical metrics, including consciousness severity and long-term functional outcomes, separately for resting and stimulated states. A total of 8 independent correlation tests were conducted, all of which were included in Benjamini–Hochberg FDR correction to avoid inflated Type I error.

Nested binary logistic regression models were constructed to evaluate the incremental prognostic value of oscillatory asymmetry for 6-month functional recovery. Five hierarchical models were established sequentially: Model 1 incorporated only conventional clinical covariates (age, duration of DOC, etiology, and CRS-R score) as the baseline clinical model; Model 2 and Model 3 added resting-state and stimulated parietal asymmetry to clinical variables, respectively; Model 4 integrated resting frontal and parietal asymmetry based on clinical covariates; Model 5 incorporated auditory-evoked frontal and parietal asymmetry indices. Receiver operating characteristic (ROC) analyses were performed to quantify model discriminatory performance. The optimal cutoff value was determined by maximizing Youden’s index, with corresponding sensitivity, specificity, positive predictive value, and negative predictive value calculated at optimal thresholds. Odds ratios (ORs) with 95% confidence intervals (CIs) and corresponding *p* values were reported for all predictive variables. To rigorously assess the generalizability and reduce potential overfitting of the final optimal model (Model 5), leave-one-out cross-validation (LOOCV) was performed for internal validation using the primary training cohort. The optimal threshold determined by LOOCV was fixed and further applied to an independent external cohort for external validation. Model performance across in-sample fitting, internal cross-validation, and external validation was comprehensively evaluated via area under the curve (AUC), sensitivity, specificity, positive predictive value, negative predictive value, and Youden’s index.

## 3. Results

### 3.1. Comparison of Baseline Information Among Three Groups

This study enrolled 57 patients with DOC, including 28 patients in the VS group and 29 patients in the MCS group, together with 23 healthy controls. Stratified Shapiro–Wilk tests confirmed that all continuous variables (age, duration of DOC, CRS-R score, and GOS score) deviated from normal distribution (all *p* < 0.05). Accordingly, intergroup comparisons of continuous variables were performed using the Mann–Whitney U test, and results were presented as median (25th, 75th percentile). 

No significant differences in age (*p* = 0.117) or sex distribution (*p* = 0.176) were detected among the three groups. In the patient subgroups, the median age was 65.00 (51.00, 73.75) years in the VS group and 67.00 (54.00, 73.50) years in the MCS group (*p* = 0.797). The median duration since brain injury was 33.50 (30.00, 44.50) days for VS patients and 40.00 (30.00, 60.00) days for MCS patients (*p* = 0.254). Lesion topography and etiological distribution were also comparable between the two DOC subgroups, with no significant intergroup differences (*p* = 0.278; *p* = 0.549, Fisher’s exact test).

Significant group disparities were exclusively observed for clinical behavioral and prognostic scores. MCS patients exhibited significantly higher CRS-R and GOS scores than VS patients. The median CRS-R score was 13.00 (8.50, 16.00) in the MCS group versus 3.50 (2.00, 5.00) in the VS group (*p* < 0.001). Correspondingly, the median GOS score was 3.00 (3.00, 4.00) for MCS patients and 2.00 (2.00, 3.00) for VS patients (*p* < 0.001). Demographic and clinical characteristics across groups are summarized in Table 1.

### 3.2. Frontoparietal Asymmetry Analysis: Global Fixed-Effect Tests

**Figure 1:**
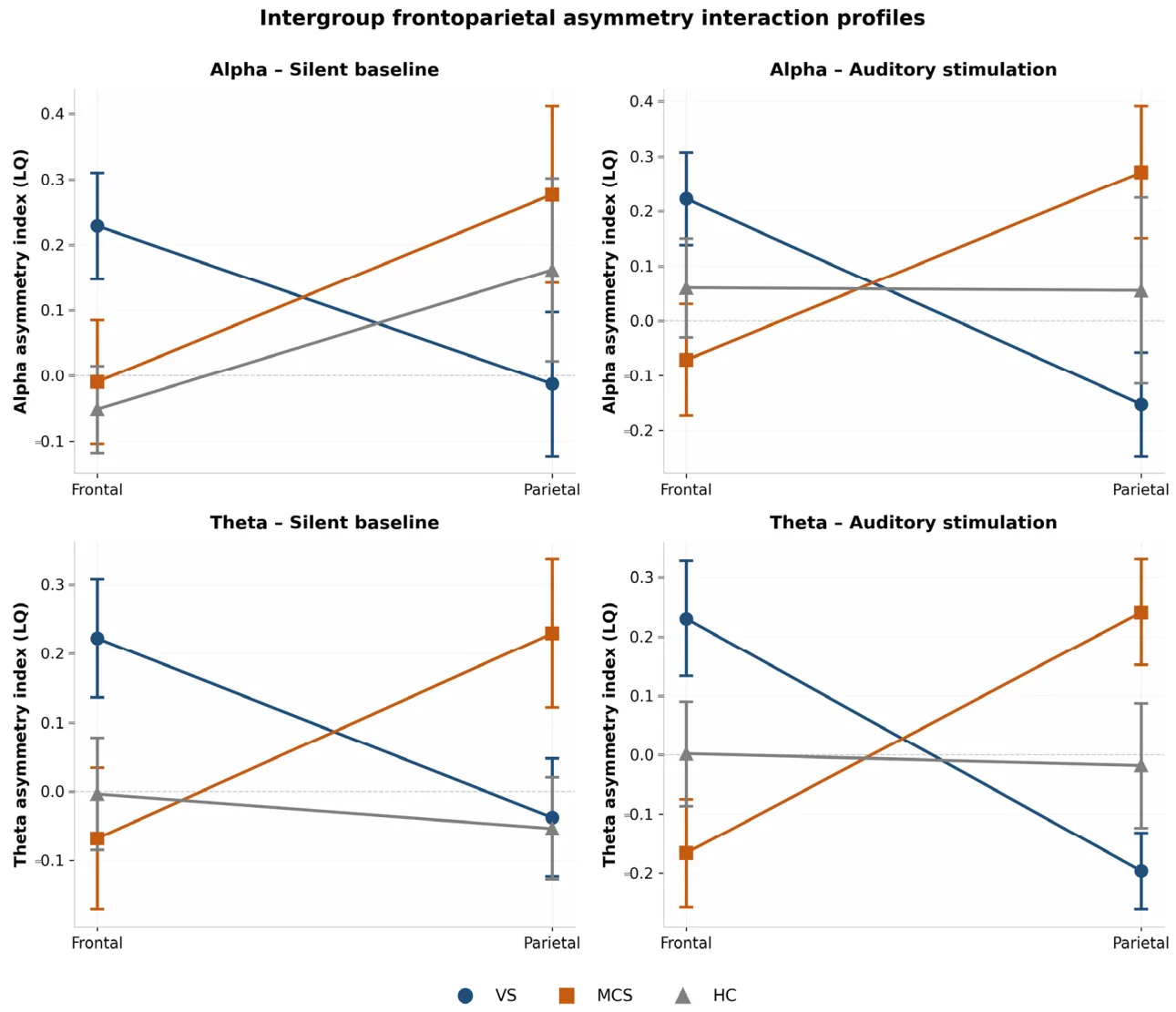
Intergroup frontoparietal asymmetry interaction profiles for the alpha (top row) and theta (bottom row) bands under silent baseline (left column) and auditory stimulation (right column). VS patients displayed elevated frontal asymmetry and reduced parietal asymmetry, whereas MCS patients demonstrated the reverse pattern; healthy controls showed near-symmetric oscillations across both regions and conditions. Blue circles, orange squares, and grey triangles correspond to VS, MCS, and HC groups, respectively. Vertical error bars represent SEM.

Age exerted a significant main effect on alpha-band oscillatory asymmetry (*F* = 5.002, *p* = 0.028), while only a marginal trend was observed for theta-band asymmetry (*F* = 3.549, *p* = 0.063). No significant main effects of sex, diagnostic group, auditory stimulation, or brain region were identified in either frequency band. A prominent diagnostic group × brain region interaction was detected for both alpha (*F* = 11.317, *p* < 0.001) and theta oscillations (*F* = 18.484, *p* < 0.001). All remaining two-way and three-way interaction terms failed to reach statistical significance for both bands (all *p* > 0.05). Full Type III fixed-effect outputs are summarized in Table 2. Figure 1 plots group means with standard errors of the mean (SEM) error bars.

### 3.3. Stratified Post Hoc Pairwise Comparisons

**Figure 2:**
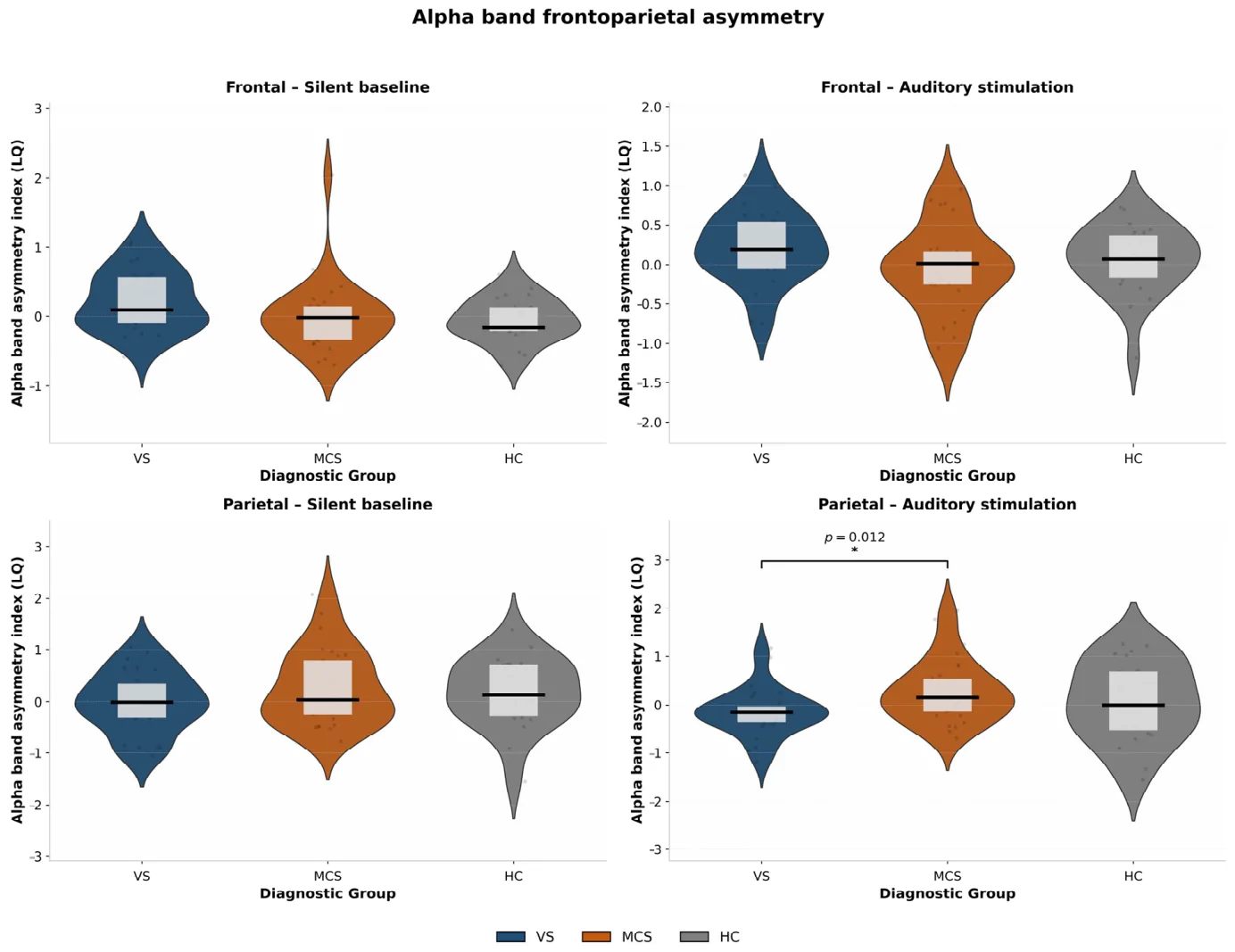
Intergroup comparison of alpha band frontal and parietal asymmetry indices under silent baseline and auditory stimulation. Each violin plot displays the kernel density estimate, with overlaid jittered individual data points, median (central horizontal line), and interquartile range (inner box). Brackets indicate significant VS–MCS pairwise comparisons with corrected *p*-values. * *p* < 0.05.

Stratified post hoc pairwise comparisons revealed distinct frequency-specific frontoparietal oscillatory asymmetry patterns between DOC subtypes at rest and during auditory stimulation. At baseline, no significant frontal or parietal asymmetry differences were found among the VS, MCS and healthy control groups in the alpha band. Following auditory stimulation, significant subgroup divergence emerged only in the parietal region: VS patients exhibited markedly lower parietal alpha asymmetry compared with MCS patients (estimate = −0.432, *p* = 0.012) (Figure 2, Table 3).

**Figure 3:**
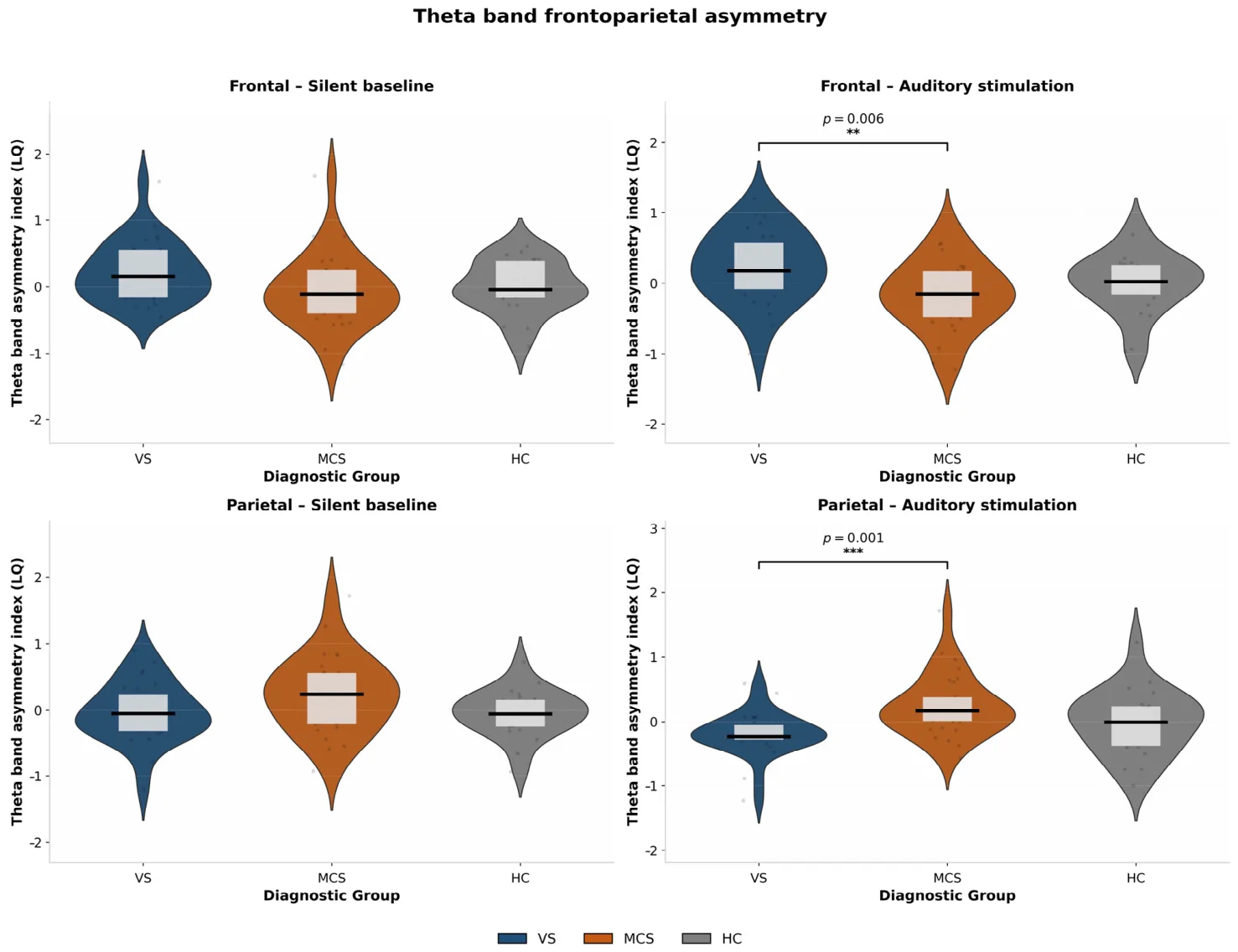
Intergroup comparison of theta band frontal and parietal asymmetry indices under silent baseline and auditory stimulation. Each violin plot displays the kernel density estimate, with overlaid jittered individual data points, median (central horizontal line), and interquartile range (inner box). Brackets indicate significant VS–MCS pairwise comparisons with corrected *p*-values. ** *p* < 0.01; *** *p* < 0.001.

In the theta band, only marginal trends were detected between VS and MCS patients at baseline: a weak positive trend in frontal asymmetry (*p* = 0.076) and a weak negative trend in parietal asymmetry (*p* = 0.079). Auditory stimulation induced prominent subtype-specific theta-band dissociation across frontoparietal regions. Relative to MCS patients, VS patients displayed significantly increased frontal theta asymmetry (estimate = 0.385, *p* = 0.006) and decreased parietal theta asymmetry (estimate = −0.447, *p* = 0.001). No significant alpha- or theta-band asymmetry differences were observed between either DOC subgroup and healthy controls under any experimental condition (Figure 3, Table 4).

### 3.4. Within-Group Stimulation Contrasts

No significant changes in alpha-band oscillatory asymmetry were detected between baseline and auditory stimulation across all diagnostic subgroups and brain regions (all adjusted *p* > 0.05). Similarly, theta-band asymmetry exhibited no meaningful state-dependent shifts between the two experimental conditions within the VS, MCS, and healthy control groups for frontal and parietal regions alike (all adjusted *p* > 0.05). This consistent null within-group effect indicates auditory stimulation does not alter intrinsic frontoparietal oscillatory balance within a single diagnostic subtype; instead, it amplifies pre-existing inter-subtype hemispheric asymmetry gaps masked at rest.

### 3.5. Inter-Regional Contrasts

At baseline, VS patients showed a marginal trend toward higher frontal alpha-band asymmetry relative to parietal asymmetry (*p* = 0.072), whereas MCS patients presented significantly greater parietal alpha asymmetry (*p* = 0.030); no inter-regional alpha-band differences were found in healthy controls. Under auditory stimulation, frontal alpha asymmetry increased significantly in the VS group (*p* = 0.005), and MCS patients maintained significantly elevated parietal alpha asymmetry (*p* = 0.010). At baseline, theta-band analyses revealed significantly higher frontal asymmetry in VS patients (*p* = 0.025) and significantly greater parietal asymmetry in MCS patients (*p* = 0.009), with no inter-regional theta-band differences observed in healthy controls. Following auditory stimulation, the frontal predominance in VS patients and parietal predominance in MCS patients were further strengthened (VS: *p* < 0.001; MCS: *p* < 0.001). Healthy controls demonstrated no frontal–parietal asymmetry imbalance in either frequency band under any experimental condition. Full inter-regional comparison results for alpha and theta bands are summarized in Table 5.

### 3.6. Spearman Correlation Analysis

**Figure 4:**
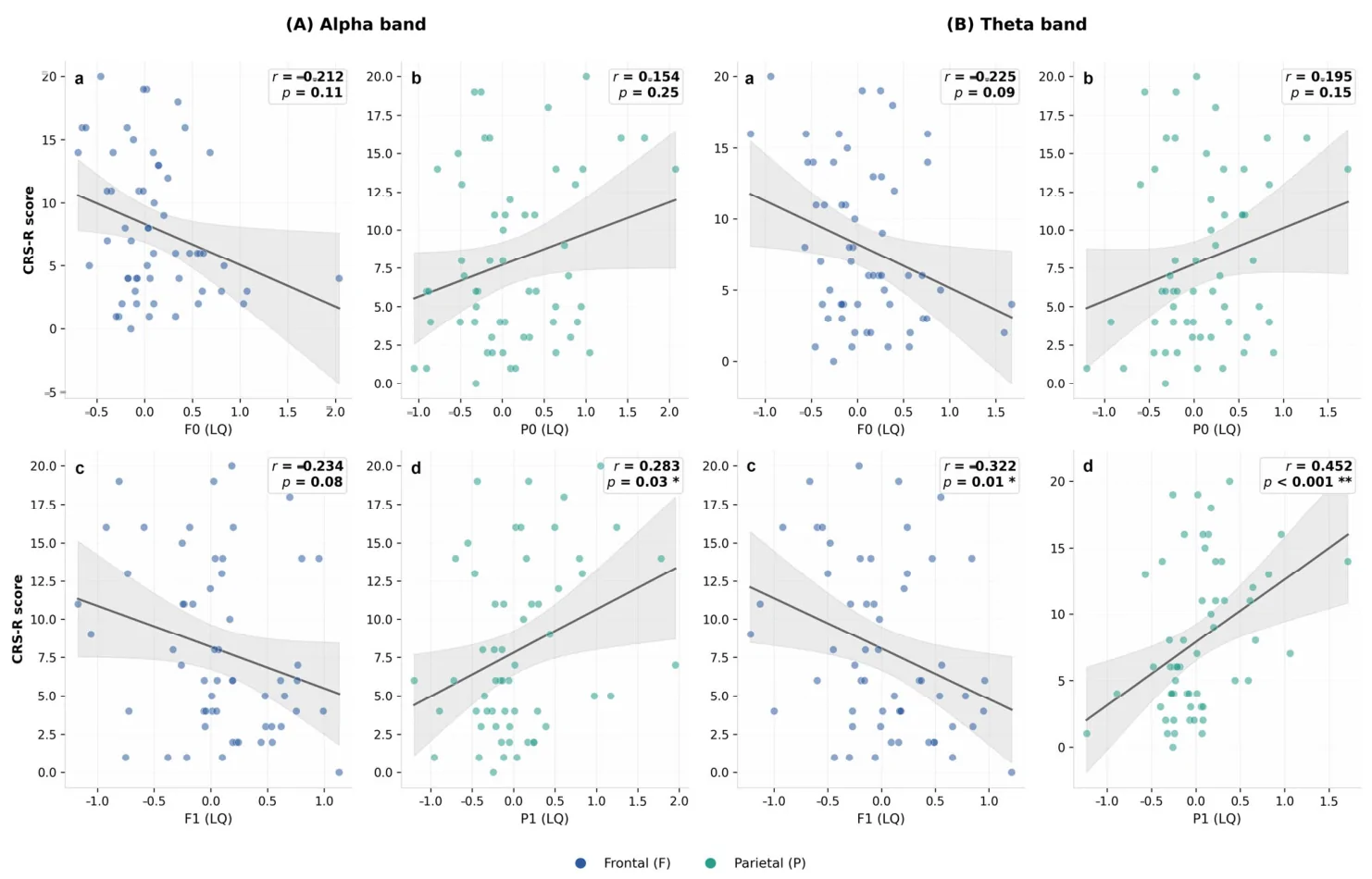
Correlation between frontoparietal asymmetry index and CRS-R score. (**A**) Alpha band; (**B**) Theta band. Solid lines represent linear regression fits, and shaded areas indicate 95% confidence intervals. Blue dots, frontal asymmetry; green dots, parietal asymmetry. *r*, Spearman correlation coefficient. Unadjusted *p* values are reported. F0, Frontal asymmetry at baseline; F1, Frontal asymmetry under auditory stimulation; P0, Parietal asymmetry at baseline; P1, Parietal asymmetry under auditory stimulation. Within each band, panels a–d correspond to F0, P0, F1, and P1, respectively. * *p* < 0.05; ** *p* < 0.01.

Following Benjamini–Hochberg multiple comparison correction, no significant correlations were detected between alpha-band asymmetry indices and CRS-R scores. Likewise, baseline theta-band asymmetry displayed no significant associations with acute consciousness status (all adjusted *p* > 0.05). Significant correlations were confined solely to theta-band metrics recorded during auditory stimulation. Specifically, stimulated frontal theta asymmetry exhibited a moderate negative correlation with CRS-R scores (*r* = −0.322, adjusted *p* = 0.029), while stimulated parietal theta asymmetry showed a strong positive correlation with CRS-R scores (*r* = 0.452, adjusted *p* = 0.002). All correlation results are illustrated in Figure 4.

### 3.7. Correlations Between Asymmetry Indices and Long-Term Prognostic Outcomes

**Figure 5:**
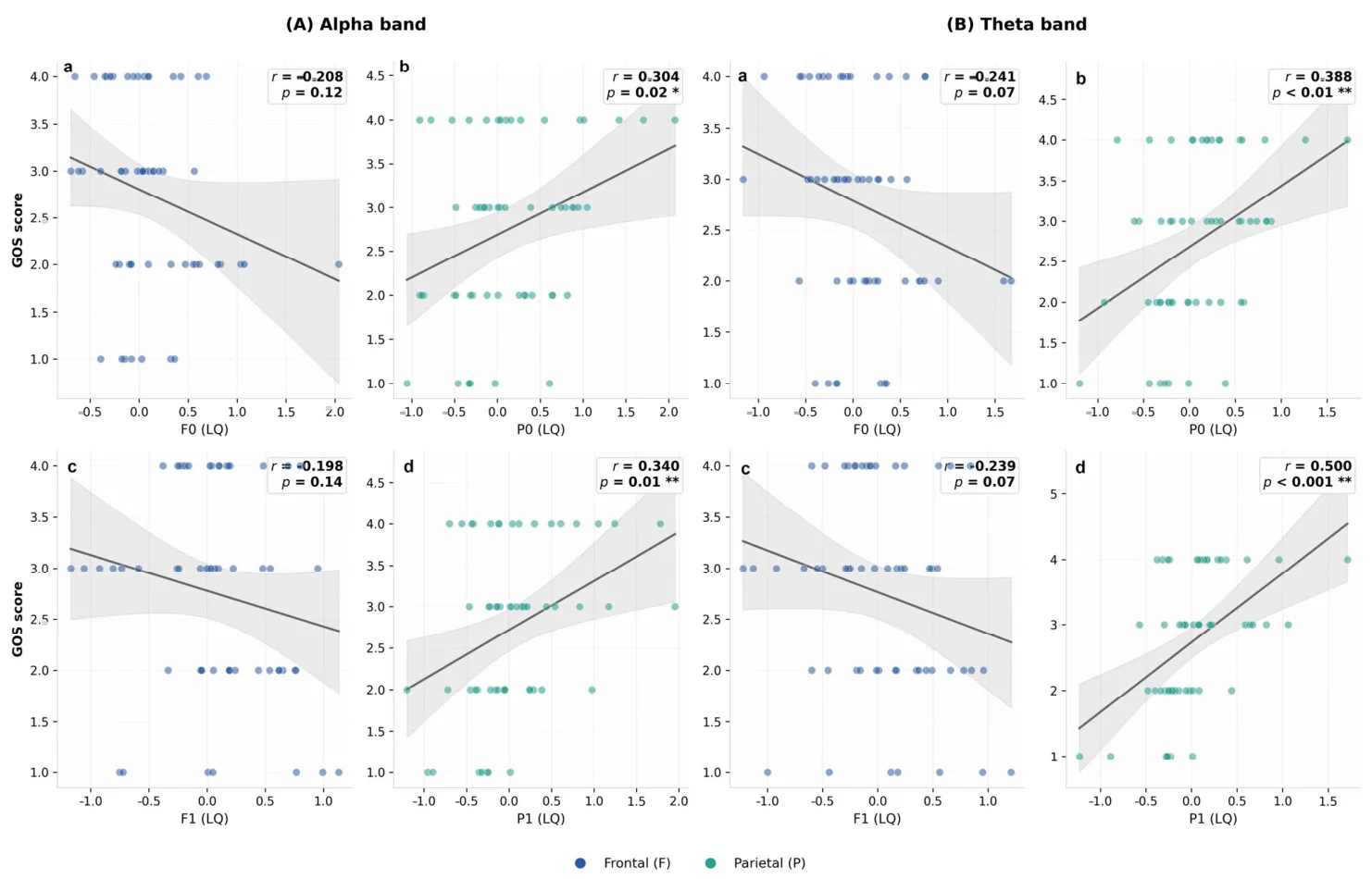
Correlation between frontoparietal asymmetry index and GOS score. (**A**) Alpha band; (**B**) Theta band. Solid lines represent linear regression fits, and shaded areas indicate 95% confidence intervals. Blue dots, frontal asymmetry; green dots, parietal asymmetry. *r*, Spearman correlation coefficient. Unadjusted *p* values are reported. F0, Frontal asymmetry at baseline; F1, Frontal asymmetry under auditory stimulation; P0, Parietal asymmetry at baseline; P1, Parietal asymmetry under auditory stimulation. Within each band, panels a–d correspond to F0, P0, F1, and P1, respectively. * *p* < 0.05; ** *p* < 0.01.

Following Benjamini–Hochberg correction, frontal oscillatory asymmetry in the alpha and theta bands demonstrated no significant linear correlations with GOS scores (all adjusted *p* > 0.05). Within the alpha band, baseline parietal asymmetry (P0, *r* = 0.304, adjusted *p* = 0.040) and stimulated parietal asymmetry (P1, *r* = 0.340, adjusted *p* = 0.040) were positively correlated with GOS scores. For the theta band, significant positive correlations were observed for both baseline parietal asymmetry (P0, *r* = 0.388, adjusted *p* = 0.010) and stimulated parietal asymmetry (P1, *r* = 0.500, adjusted *p* < 0.01). All correlation results are illustrated in Figure 5.

### 3.8. Logistic Regression Prognostic Modeling

**Figure 6:**
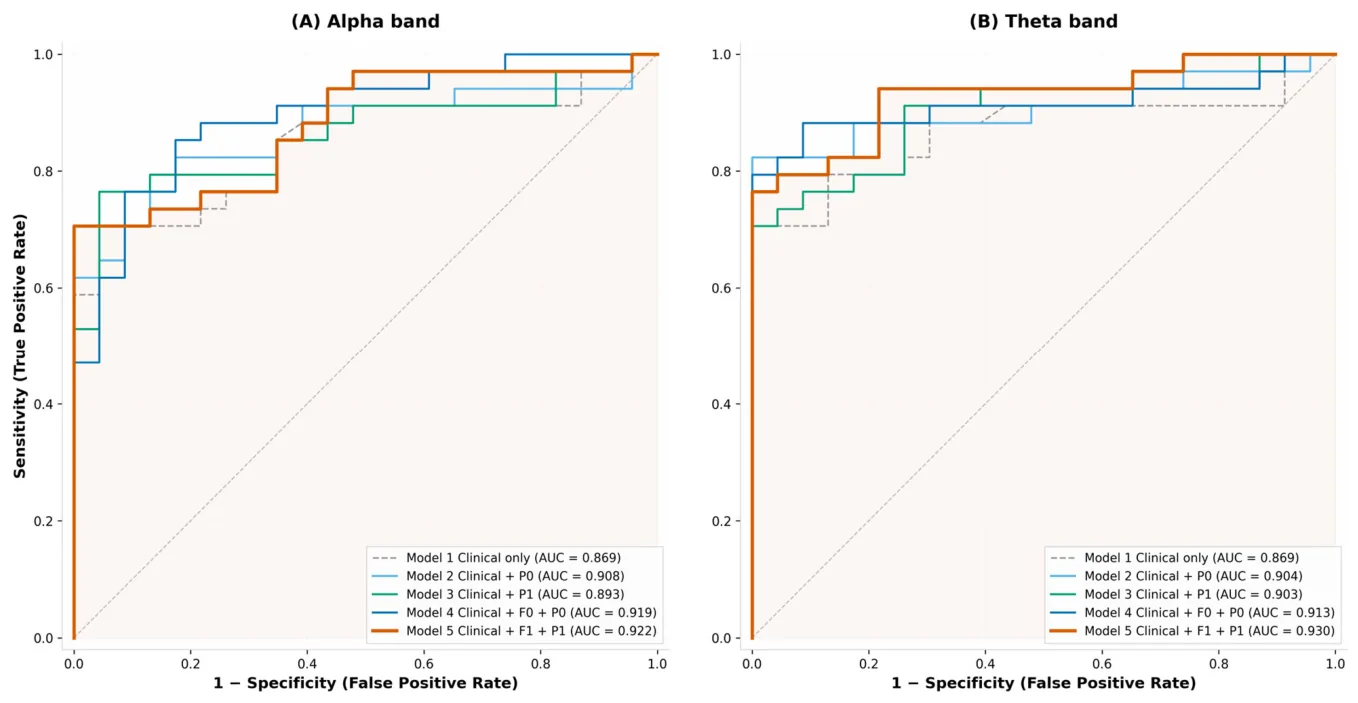
ROC analyses of nested prognostic models for 6-month functional outcome prediction. (**A**) Alpha band; (**B**) Theta band. Solid lines represent fitted ROC curves for five hierarchically nested models; the dashed gray line indicates the reference diagonal (AUC = 0.500). Model 1, clinical covariates only; Model 2, clinical + baseline parietal asymmetry (P0); Model 3, clinical + post-stimulation parietal asymmetry (P1); Model 4, clinical + baseline frontal (F0) and parietal (P0) asymmetry; Model 5, clinical + post-stimulation frontal (F1) and parietal (P1) asymmetry. Areas under the curve (AUC) are reported in the legend. F0, Frontal asymmetry at baseline; F1, Frontal asymmetry under auditory stimulation; P0, Parietal asymmetry at baseline; P1, Parietal asymmetry under auditory stimulation.

Diagnostic and predictive metrics for all nested prognostic models are summarized in Table 6. Relative to the clinical-only baseline model, all models incorporating frontoparietal oscillatory asymmetry indices exhibited improved model fitting and discriminatory capacity, with consistently superior prognostic performance. Among all established models, theta-band Model 5 represented the optimal predictive paradigm. This hierarchical model integrated conventional clinical covariates with auditory stimulation-evoked frontal and parietal asymmetry indices, yielding a maximum AUC of 0.930. Similarly, alpha-band Model 5 achieved favorable predictive efficacy with an AUC of 0.922 (Table 6, Figure 6).

Likelihood ratio tests were further conducted to evaluate incremental model fit across nested alpha- and theta-band asymmetry models. In the alpha band, the addition of baseline parietal asymmetry (P0) significantly improved the fit of the baseline clinical model (*χ*^2^ = 4.416, *p* = 0.036), whereas post-stimulation parietal asymmetry (P1) only yielded a marginal, non-significant improvement (*χ*^2^ = 3.011, *p* = 0.083). In contrast, the incorporation of baseline and post-stimulation frontal asymmetry indices (F0, F1) produced significant incremental improvements in model fit (*p* = 0.031 and *p* = 0.016, respectively), confirming the independent supplementary predictive value of frontal asymmetry beyond parietal oscillatory metrics. For the theta band, both baseline and post-stimulation parietal asymmetry (P0, P1) significantly enhanced the performance of the clinical baseline model (*p* = 0.013, *p* = 0.015). However, frontal asymmetry provided limited supplementary predictive power: baseline frontal asymmetry (F0) contributed no significant model improvement (*p* = 0.143), while post-stimulation frontal asymmetry (F1) only showed a marginal trending effect (*p* = 0.061).

We further assessed the generalizability and robustness of the optimal theta-band Model 5 using leave-one-out cross-validation (LOOCV) and an independent temporal external cohort. LOOCV yielded an AUC of 0.857, with a sensitivity of 76.5% and specificity of 82.6%. The optimal threshold was determined by maximizing Youden’s index based on the LOOCV results of the derivation cohort and then applied to the external validation cohort. In the external cohort, the model attained an AUC of 0.904, a sensitivity of 65.2%, and a specificity of 92.9%. The predictive performance metrics across in-sample fitting, internal cross-validation, and external validation are summarized in Table 7.

## 4. Discussion

The present study systematically characterized frontoparietal interhemispheric oscillatory asymmetry in DOC patients under resting baseline and auditory stimulated states, yielding three key findings. First, a robust diagnostic group × brain region interaction was identified across both alpha and theta bands. VS patients exhibited frontal-dominant asymmetry, whereas MCS patients showed parietal-dominant asymmetry; this dissociable electrophysiological phenotype was markedly amplified by auditory stimulation. Second, only auditory-evoked theta-band asymmetry was significantly correlated with consciousness severity and long-term functional recovery. Third, the integration of stimulated frontoparietal theta asymmetry substantially improved prognostic discrimination relative to conventional clinical models alone, with the optimal nested logistic model achieving an AUC of 0.930. Collectively, auditory stimulation triggered frontoparietal theta asymmetry represents a frequency- and state-dependent biomarker for DOC subtyping and medium-term functional outcome prediction.

Importantly, the asymmetry indices in the present study were derived from two homologous frontal and parietal electrode pairs. Therefore, they should be interpreted as regional, electrode-level markers of interhemispheric oscillatory imbalance rather than direct measures of whole-brain frontoparietal network organization or connectivity. Network-level interpretations discussed below are informed by convergent evidence from previous neurophysiological and neuroimaging studies and should therefore be regarded as mechanistically suggestive rather than directly demonstrated by the present electrode montage.

### 4.1. Frontoparietal Oscillatory Asymmetry as a Subtype-Specific DOC Phenotype

This study identified significant subgroup-specific frontoparietal asymmetry patterns across alpha and theta oscillations, demonstrating distinct electrophysiological phenotypes that differentiate VS and MCS. Specifically, VS patients exhibited predominant frontal interhemispheric oscillatory imbalance, while MCS patients presented dominant parietal lateralization. Notably, this spatial dissociation was substantially amplified by auditory stimulation, whereas healthy controls maintained symmetric frontoparietal oscillatory patterns without lateral bias at rest. At baseline, DOC subtype differences were limited to marginal theta-band trends and negligible alpha-band discrepancies. In contrast, auditory stimulation unmasked robust frontal and parietal asymmetry disparities between VS and MCS cohorts, with theta oscillations exhibiting the most prominent subtype differentiation. We caution, however, that healthy controls were somewhat younger than DOC patients, and age exerted a significant main effect on alpha-band asymmetry. Although age and sex were statistically adjusted as covariates in all linear mixed-effects models, this demographic imbalance warrants consideration when interpreting the control group as a symmetry benchmark, particularly for alpha-band comparisons.

Healthy control symmetric frontoparietal oscillations provide a critical physiological reference benchmark for pathological DOC asymmetry. Intact interhemispheric frontoparietal balance is the electrophysiological foundation of integrated conscious information processing under normal physiological conditions; any directional lateral shift reflects partial disruption of bilateral frontoparietal synchronization. The universal symmetry observed in healthy participants further verifies that frontal-dominant or parietal-dominant hemispheric imbalance is a disease-specific electrophysiological trait exclusive to DOC patients.

These spatial dissociation patterns align with prior neurophysiological and neuroimaging evidence. Graph-based resting-state EEG studies have confirmed VS-specific interhemispheric frontoparietal disconnection, corroborating the spatially differentiated asymmetry profiles observed in the present study [5]. Functional neuroimaging further supports this pathophysiological framework: frontoparietal functional decoupling correlates linearly with consciousness severity, and hemispheric imbalance provides diagnostic value independent of global spectral power metrics [6,25]. Conceptually, the present qEEG asymmetry indices mirror these neuroimaging-derived decoupling patterns at the electrophysiological level. In the healthy brain, symmetric frontoparietal oscillatory activity reflects balanced interhemispheric information exchange within the global neuronal workspace. In DOC, however, the breakdown of this symmetry—manifested as increased frontal and/or parietal asymmetry in theta and alpha oscillations—parallels the reduced frontoparietal functional connectivity observed in fMRI and PET studies. Specifically, our finding that theta-band parietal asymmetry (P1) correlates positively with CRS-R scores and 6-month GOS aligns with the neuroimaging observation that posterior parietal hub dysfunction is a hallmark of impaired awareness. Likewise, the elevated frontal asymmetry characteristic of VS patients in our sample corresponds to the severe disruption of executive-control network nodes documented with functional neuroimaging. It is important to emphasize that electrode-level asymmetry and network-level functional connectivity represent distinct but complementary measures: whereas neuroimaging captures slow hemodynamic correlations across distributed voxels, qEEG asymmetry captures instantaneous oscillatory power imbalances between homologous regions. The convergent findings across these modalities nonetheless substantiate a common pathophysiological theme—namely, that disruption of frontoparietal interhemispheric balance constitutes a robust, modality-independent marker of consciousness impairment. Repetitive transcranial magnetic stimulation (rTMS) studies have additionally verified inherent regional lateralization deficits in DOC patients, wherein pretreatment pathological asymmetry diminishes following effective neuromodulation [26]. Symmetric frontoparietal oscillatory activity under resting and stimulated conditions is exclusively observed in healthy individuals, confirming hemispheric imbalance as a disease-specific electrophysiological trait. Collectively, region-specific frontoparietal oscillatory asymmetry serves as a valid phenotypic marker for distinguishing DOC subtypes.

The present hemispheric asymmetry analyses further refine subtype-specific frontoparietal lateralization signatures. MCS patients with residual conscious capacity exhibit elevated parietal asymmetry and relatively reduced frontal asymmetry, reflecting preserved integrity of left frontal and right parietal conscious processing circuits. In contrast, patients with dominant frontal asymmetry predominantly meet diagnostic criteria for VS, consistent with severe functional impairment within core frontoparietal conscious hubs. Neuroimaging evidence elucidates the underlying biological basis: the posterior parietal cortex and precuneus serve as pivotal nodes for awareness-related frontoparietal networks and the default mode network. Metabolic activity within these regions couples tightly with thalamic function and recovers progressively alongside clinical improvement. During passive auditory stimulation, healthy individuals and partial MCS patients exhibit adaptive medial frontoparietal deactivation indicative of external cognitive reorientation, a regulatory response absent in VS patients. Task-induced frontoparietal deactivation typically coincides with left frontal activation, a well-established correlate of preserved high-order cognitive processing [27].

These findings are further corroborated by our previous high-definition transcranial direct current stimulation (HD-tDCS) investigations. Theta-band phase locking value (PLV) across left frontoparieto-occipital regions positively correlates with Coma Recovery Scale-Revised (CRS-R) improvements. Responsive patients demonstrate enhanced theta PLV and elevated frontal alpha PLV following repeated stimulation, whereas non-responders show reduced frontal alpha PLV without behavioral improvement [28]. This mechanistic distinction explains the superior performance of theta asymmetry for consciousness severity grading and prognostic evaluation, as residual left frontal circuit integrity is selectively preserved in MCS patients. Complementary rTMS findings reinforce this lateralization model: frontal alpha asymmetry (FAA) shifts leftward in treatment responders and rightward in non-responders, with left FAA strongly associated with milder long-term disability. rTMS-induced improvements in alpha power and network connectivity are predominantly localized to the right parietal cortex [26]. Metabolic imaging further validates this framework, confirming preserved left frontal–right parietal connectivity and weakened right frontal–left parietal coupling in DOC populations [29]. Integrative electrophysiological, neuroimaging, and neuromodulatory evidence collectively substantiates the existence of robust subtype-specific frontoparietal asymmetry phenotypes in DOC.

### 4.2. Auditory Stimulation Unmasks Latent Interregional Network Deficits

Resting-state qEEG evaluation is inherently limited by spontaneous arousal fluctuations and suppressed residual neural activity, rendering subtle intergroup network differences difficult to detect reliably. Passive auditory stimulation serves as a feasible, bedside-accessible physiological probe capable of activating latent conscious circuits and exposing functional network disruptions that remain undetectable at baseline, consistent with contemporary DOC neurophysiological research. Conscious awareness relies on integrated signal propagation across the frontoparietal neuronal workspace, and structural or functional damage to these long-range interhemispheric pathways impairs perceptual and conscious processing [30,31]. Human cognition depends on two complementary frontoparietal attentional systems requiring intact cross-hemispheric communication: the dorsal attention network mediates goal-directed voluntary attention, while the right ventral attention network governs automatic stimulus reorientation. Auditory input preferentially activates the ventral attention pathway, evoking time-locked oscillatory responses and amplifying subtype-specific lateralization differences masked under resting conditions.

Sensory perturbation has been consistently validated to unmask covert network dysfunction invisible in resting qEEG recordings [32,33,34]. Prior nonlinear qEEG studies have demonstrated that tailored musical stimuli selectively enhance cortical excitability in MCS patients, while comparable electrophysiological modulation is absent in VS cohorts [35]. Attentional resource allocation is dynamically regulated by reciprocal crosstalk between the dorsal and ventral frontoparietal attention circuits [36]. In the present cohort, theta-band intergroup differences only reached marginal significance at rest, whereas robust VS–MCS differentiation emerged following auditory stimulation. Intra-individual pre–post comparisons excluded universal oscillatory alterations across all participants, confirming that auditory stimulation exerts divergent regulatory effects on frontal and parietal cortical activity according to DOC subtype. These findings explain the limited discriminative power of conventional resting spectral analyses and highlight the necessity of stratified LMMs for resolving complex multi-way interactions. Collectively, these results validate the physiological probe hypothesis: external sensory perturbation efficiently exposes latent frontoparietal network heterogeneity that is absent in resting EEG signals.

Finally, all participants received the same generic musical excerpt. This standardization ensured identical acoustic properties, duration, intensity, and delivery across groups and states, supporting the interpretation that the observed VS–MCS dissociation reflects differential responses to a common auditory probe. Nevertheless, this design did not capture individual differences in musical familiarity, preference, emotional salience, or autobiographical relevance. Familiar or personally meaningful stimuli might elicit stronger responses, particularly in MCS patients, whereas an unfamiliar excerpt could attenuate or increase the variability of these responses. The present effects should therefore be regarded as responses to standardized auditory stimulation rather than maximal individualized responses. Although this may have underestimated the true effect sizes, significant VS–MCS differences despite stimulus standardization support the robustness of the identified theta-asymmetry phenotype. Future within-subject studies should directly compare standardized and personalized stimuli while prospectively assessing familiarity, preference, and emotional salience.

### 4.3. Frequency Specificity: Theta Versus Alpha

The present study identified distinct frequency-specific dissociation patterns, wherein only auditory-evoked theta-band asymmetry correlated significantly with both CRS-R scores and long-term GOS outcomes. This selective functional specificity stems from divergent physiological roles of alpha and theta oscillations within frontoparietal conscious circuits. Alpha oscillations primarily subserve top-down sensory gating during wakefulness, mediating predictive suppression of redundant sensory inputs via high-order cortical modulation. During pharmacologically induced unconsciousness, this hierarchical regulatory pathway is disrupted, characterized by diminished frontosensory alpha coherence, impaired temporal alpha modulation of auditory signals, and loss of structured cognitive processing [37]. In contrast, theta oscillations sustain distributed frontoparietal network integration, which is indispensable for conscious awareness. Theta-band activity is consistently validated as a reliable biomarker of residual thalamo-cortical functional integrity following severe brain injury [38,39].

Longitudinal clinical studies further support this frequency-dependent functional hierarchy. Serial CRS-R fluctuations correlate most strongly with theta power and alpha connectivity metrics, with early EEG biomarkers outperforming single time-point behavioral assessments for prognostic prediction [40]. The present findings extend this framework by demonstrating that theta-band frontoparietal asymmetry is more closely associated with consciousness severity and long-term functional recovery, attributable to theta’s superior sensitivity to post-injury network reorganization [36]. Multimodal qEEG evidence corroborates this conclusion: robust MCS–VS differentiation is observed in theta spatiotemporal entropy across cortical regions, with theta indices enabling regional discrimination across frontal, parietal, temporal, and occipital cortices, whereas alpha biomarkers exhibit limited posterior regional discriminative capacity. Auditory stimulation further amplifies parieto-occipital theta-band subtype disparities [41]. Collectively, theta oscillations capture refined signatures of frontoparietal integrative circuit impairment, providing superior discriminative performance for consciousness evaluation and prognostic modeling in DOC.

### 4.4. Incremental Prognostic Utility of Stimulus-Induced Oscillatory Asymmetry

Timely and accurate prediction of long-term functional recovery remains a critical unmet clinical need in DOC management. Conventional behavioral rating scales are susceptible to fluctuating arousal levels and covert residual consciousness, while traditional prognostic models rarely incorporate dynamic neurophysiological biomarkers to enhance predictive accuracy [42,43]. In this study, nested binary logistic regression analyses confirmed that integrating frontoparietal oscillatory asymmetry metrics significantly improved model discriminatory performance. Among all evaluated biomarkers, auditory-evoked theta asymmetry yielded the highest AUC values. Likelihood ratio tests further verified that parietal theta asymmetry provides independent incremental prognostic value beyond routine clinical covariates, with superior predictive efficacy confirmed via ROC comparative analysis.

Longitudinal neuromodulation studies provide convergent prognostic validation. Baseline parietal alpha asymmetry and frontal alpha synchrony asymmetry reliably predict 12-month functional disability in VS patients. Consistent with our results, rTMS investigations confirm that patients with right-lateralized parietal alpha asymmetry tend to achieve favorable long-term functional outcomes [26]. Furthermore, multimodal EEG predictive modeling supports the translational value of this stimulation paradigm. Combined resting-state nonlinear features and auditory event-related potential (ERP) markers enable rapid, objective bedside differentiation between MCS and VS patients, minimizing subjective bias inherent to behavioral CRS-R assessments [41]. Collectively, these findings demonstrate that auditory stimulation-modulated EEG signatures constitute robust, noninvasive biomarkers for precise DOC subtyping and reliable long-term prognostic stratification.

Our subsequent internal leave-one-out cross-validation and temporal external cohort validation further confirmed the robustness and generalizability of the optimal theta-band prognostic model (Model 5). The model maintained good predictive performance in LOOCV (AUC = 0.857). In the temporally independent external cohort from the same hospital, the model achieved an AUC of 0.904, with slightly reduced sensitivity but preserved high specificity. The slight drop in sensitivity in the external cohort may partly reflect differences in patient clinical profiles between derivation and validation samples, as well as the relatively small sample size of the external validation set. These validation results support the potential clinical utility of theta asymmetry as a prognostic biomarker, though multi-center external validation is still required for broader clinical application.

### 4.5. Translational Implications for Personalized Non-Invasive Neuromodulation

The subtype-specific frontoparietal asymmetry patterns identified in this study carry important clinical implications for individualized non-invasive brain stimulation (NIBS) rehabilitation. Current DOC neuromodulation trials exhibit highly variable therapeutic responses, largely attributable to uniform empirical stimulation protocols without physiology-based patient stratification. Mechanistically, neuromodulation-induced clinical improvements rely on the restoration of interhemispheric oscillatory balance and frontoparietal network synchronization. Previous rTMS studies have shown that VS patients responsive to left parietal stimulation exhibit adaptive leftward frontal alpha lateralization accompanied by behavioral recovery, whereas non-responders fail to achieve such changes in regional oscillatory asymmetry, indicating frontoparietal functional asymmetry as a key physiological determinant of treatment efficacy [26].

Instead of directly resolving heterogeneous treatment outcomes, our asymmetry phenotype framework provides a theoretical stratification reference for future prospective NIBS interventional trials. Accordingly, our findings support an asymmetry-tailored NIBS strategy for DOC intervention. For VS patients characterized by severe frontal functional imbalance and dominant frontal asymmetry, targeted parietal stimulation may enhance residual parietal circuit activity and reconstruct interhemispheric frontoparietal coupling and network integration within this circuit. For MCS patients with relatively preserved parietal function and parietal-dominant asymmetry, prefrontal neuromodulation may further facilitate high-order conscious information processing. Furthermore, theta asymmetry biomarkers enable both baseline phenotypic stratification and dynamic longitudinal monitoring of regional oscillatory reorganization during intervention. This phenotype-guided individualized paradigm addresses heterogeneous treatment responses in unselected DOC cohorts, advancing DOC neuromodulation from empirical intervention toward precision, personalized rehabilitation therapy.

### 4.6. Novelty and Comparison with Existing Literature

Prior DOC qEEG studies lack systematic comparison of resting and auditory-evoked frontoparietal asymmetry across distinct frequency bands. Most existing investigations focus on spectral power or functional connectivity rather than interhemispheric oscillatory balance. The present work establishes an integrated analytical pipeline encompassing subtype differentiation, clinical correlation analysis, and multivariable prognostic modeling, providing a standardized, replicable framework for future DOC neurophysiological research.

## 5. Limitations

This study has several limitations. First, this study adopted a single-center, inpatient-only design. Although we performed temporal external validation using 37 independent DOC patients recruited during a subsequent enrollment period with identical inclusion criteria and assessment protocols, the generalizability of our findings remains limited. Case-mix characteristics and clinical management patterns in our tertiary hospital cohort may differ from those of general hospitals or rehabilitation populations. Therefore, further multi-center prospective external validation is warranted to verify the broader applicability of this theta-asymmetry-based prognostic model. Second, only frontal and parietal electrodes were analyzed; whole-brain EEG mapping covering temporal, occipital and subcortical-associated cortical regions is needed for comprehensive phenotype characterization. Third, all participants received the same generic musical excerpt. This standardized stimulus guaranteed consistent acoustic parameters across groups, yet it failed to account for individual variation in musical familiarity, preference, emotional salience, and autobiographical relevance. Personalized familiar sounds may evoke stronger neural responses, so our observed effects represent conservative estimates of the true response magnitude. Fourth, healthy controls were younger than DOC patients, and age showed a significant main effect on alpha-band asymmetry. Although age and sex were included as covariates in all statistical models, this demographic imbalance limits the direct interpretability of the control group as an age-matched symmetry benchmark, particularly for alpha-band analyses.

## 6. Conclusions

VS and MCS exhibit distinct frontoparietal interhemispheric oscillatory asymmetry phenotypes that are selectively amplified by auditory stimulation, particularly within the theta band. Stimulus-evoked theta frontoparietal asymmetry robustly reflects the severity of conscious dysfunction and provides incremental prognostic value for early prediction of long-term functional recovery. Collectively, this study establishes a validated neurophysiological biomarker and translational framework to support accurate DOC subtyping, early prognostication, and precision rehabilitation intervention.

## Figures and Tables

**Table 1:** Baseline Demographic and Clinical Profiles of Patients With Disorders of Consciousness and Healthy Controls.

Variables	VS (*n* = 28)	MCS (*n* = 29)	HC (*n* = 23)	Mann–Whitney *U* Test/*χ*^2^ Test/Fisher’s Exact Test	*p*
Age (years)	65 (51, 73.75)	67 (54, 73.5)	60 (32, 68)	4.283	0.117
Sex, *n* (%)				3.480	0.176
Female	7 (25.00%)	13 (44.83%)	11 (47.83%)		
Male	21 (75.00%)	16 (55.17%)	12 (52.17%)		
Duration of DOC (days)	33.50 (30.00, 44.50)	40.00 (30.00, 60.00)	-	1.140	0.254
CRS-R score	3.50 (2.00, 5.00)	13.00 (8.50, 16.00)	-	6.131	<0.001
GOS score	2.00 (2.00, 3.00)	3.00 (3.00, 4.00)	-	4.038	<0.001
Lesion location, *n* (%)			-	3.851	0.278
Left hemisphere	6 (21.43%)	13 (44.83%)			
Right hemisphere	5 (17.86%)	5 (17.24%)			
Brainstem region	11 (39.29%)	7 (24.14%)			
Diffuse cerebral lesions	6 (21.43%)	4 (13.79%)			
Etiology, *n* (%)			-	2.345	0.549
Cerebral infarction	9 (32.14%)	12 (41.38%)			
Cerebral hemorrhage	13 (46.43%)	13 (44.83%)			
Traumatic brain injury	4 (14.29%)	1 (3.45%)			
Hypoxic–ischemic encephalopathy	2 (7.14%)	3 (10.34%)			

VS, vegetative state; MCS, minimally conscious state; HC, healthy controls; CRS-R, Coma Recovery Scale-Revised; GOS, Glasgow Outcome Scale.

**Table 2:** Type III fixed-effect test results of frontoparietal asymmetry.

Effect	*F*-Alpha	*p*-Alpha	*F*-Theta	*p*-Theta
Age	5.002	0.028	3.549	0.063
Sex	1.326	0.253	1.753	0.189
Diagnostic group	0.211	0.810	0.089	0.914
Auditory stimulus	0.376	0.539	0.444	0.505
Brain region	0.424	0.515	0.031	0.859
Group × Stimulus	0.144	0.865	0.314	0.730
Group × Region	11.317	<0.001	18.484	<0.001
Stimulus × Region	0.767	0.381	0.008	0.926
Group × Stimulus × Region	0.524	0.592	0.757	0.469

**Table 3:** Pairwise post hoc comparisons of alpha band frontoparietal asymmetry.

Comparison	Stimulation Condition	Brain Region	Mean Difference (Estimate)	Standard Error	*t*	Adjusted *p*
VS–MCS	Silent baseline	Frontal	0.229	0.150	1.529	0.382
VS–HC	Silent baseline	Frontal	0.206	0.161	1.276	0.608
MCS–HC	Silent baseline	Frontal	−0.023	0.159	−0.143	1.000
VS–MCS	Auditory stimulation	Frontal	0.284	0.150	1.898	0.176
VS–HC	Auditory stimulation	Frontal	0.088	0.161	0.548	1.000
MCS–HC	Auditory stimulation	Frontal	−0.196	0.159	−1.229	0.660
VS–MCS	Silent baseline	Parietal	−0.298	0.150	−1.989	0.143
VS–HC	Silent baseline	Parietal	−0.248	0.161	−1.537	0.376
MCS–HC	Silent baseline	Parietal	0.049	0.159	0.311	1.000
VS–MCS	Auditory stimulation	Parietal	−0.432	0.150	−2.884	0.012
VS–HC	Auditory stimulation	Parietal	−0.281	0.161	−1.741	0.248
MCS–HC	Auditory stimulation	Parietal	0.151	0.159	0.946	1.000

VS, vegetative state; MCS, minimally conscious state; HC, healthy control. Mean difference = former group minus latter group. All *p* values were corrected via the Bonferroni method for three pairwise comparisons within each stratum.

**Table 4:** Pairwise group comparisons for theta band frontoparietal asymmetry.

Comparison	Stimulation Condition	Brain Region	Mean Difference (Estimate)	Standard Error	*t*	Adjusted *p*
VS–MCS	Silent baseline	Frontal	0.279	0.124	2.246	0.076
VS–HC	Silent baseline	Frontal	0.172	0.134	1.283	0.601
MCS–HC	Silent baseline	Frontal	−0.107	0.132	−0.814	1.000
VS–MCS	Auditory stimulation	Frontal	0.385	0.124	3.096	0.006
VS–HC	Auditory stimulation	Frontal	0.174	0.134	1.303	0.580
MCS–HC	Auditory stimulation	Frontal	−0.210	0.132	−1.593	0.336
VS–MCS	Silent baseline	Parietal	−0.277	0.124	−2.231	0.079
VS–HC	Silent baseline	Parietal	−0.038	0.134	−0.283	1.000
MCS–HC	Silent baseline	Parietal	0.239	0.132	1.812	0.213
VS–MCS	Auditory stimulation	Parietal	−0.447	0.124	−3.595	0.001
VS–HC	Auditory stimulation	Parietal	−0.232	0.134	−1.731	0.253
MCS–HC	Auditory stimulation	Parietal	0.215	0.132	1.630	0.312

VS, vegetative state; MCS, minimally conscious state; HC, healthy control. Mean difference = former group minus latter group. All *p* values were corrected by Bonferroni method for three pairwise comparisons within each stratum.

**Table 5:** Pairwise comparisons between frontal and parietal regions.

Band	Comparison	Diagnostic Group	Stimulation Condition	Mean Difference (Estimate)	Standard Error	*t*	Adjusted *p*
Alpha	Frontal–Parietal	VS	Silent baseline	0.241	0.133	1.806	0.072
Alpha	Frontal–Parietal	MCS	Silent baseline	−0.286	0.131	−2.176	0.030
Alpha	Frontal–Parietal	HC	Silent baseline	−0.213	0.147	−1.445	0.149
Alpha	Frontal–Parietal	VS	Auditory stimulation	0.375	0.133	2.804	0.005
Alpha	Frontal–Parietal	MCS	Auditory stimulation	−0.342	0.131	−2.603	0.010
Alpha	Frontal–Parietal	HC	Auditory stimulation	0.005	0.147	0.034	0.972
Theta	Frontal–Parietal	VS	Silent baseline	0.260	0.115	2.247	0.025
Theta	Frontal–Parietal	MCS	Silent baseline	−0.297	0.113	−2.618	0.009
Theta	Frontal–Parietal	HC	Silent baseline	0.050	0.127	0.392	0.695
Theta	Frontal–Parietal	VS	Auditory stimulation	0.426	0.115	3.686	<0.001
Theta	Frontal–Parietal	MCS	Auditory stimulation	−0.406	0.113	−3.580	<0.001
Theta	Frontal–Parietal	HC	Auditory stimulation	0.019	0.127	0.155	0.876

VS, vegetative state; MCS, minimally conscious state; HC, healthy control. Mean difference = frontal region minus parietal region. All *p* values were corrected using the Bonferroni method.

**Table 6:** Prognostic performance of nested prognostic models.

Band	Model	Predictor Set	AUC	Sensitivity (%)	Specificity (%)	PPV (%)	NPV (%)	Youden’s Index
Alpha	1	Clinical covariates only	0.869	70.6	100.0	100.0	69.7	0.706
Alpha	2	Clinical + P0	0.908	82.4	100.0	100.0	79.3	0.824
Alpha	3	Clinical + P1	0.893	76.5	95.7	96.3	73.3	0.721
Alpha	4	Clinical + F0 + P0	0.919	82.4	95.7	96.6	78.6	0.780
Alpha	5	Clinical + F1 + P1	0.922	76.5	100.0	100.0	74.2	0.765
Theta	1	Clinical covariates only	0.869	70.6	100.0	100.0	69.7	0.706
Theta	2	Clinical + P0	0.904	82.4	100.0	100.0	79.3	0.824
Theta	3	Clinical + P1	0.903	70.6	100.0	100.0	69.7	0.706
Theta	4	Clinical + F0 + P0	0.913	88.2	91.3	93.8	84.0	0.795
Theta	5	Clinical + F1 + P1	0.930	79.4	95.7	96.4	75.9	0.751

F0, Frontal asymmetry at baseline; F1, Frontal asymmetry under auditory stimulation; P0, Parietal asymmetry at baseline; P1, Parietal asymmetry under auditory stimulation; AUC, area under the curve; PPV, positive predictive value; NPV, negative predictive value.

**Table 7:** Internal leave-one-out cross-validation and external validation performance of the optimal theta-band prognostic model (Model 5).

Validation Strategy	AUC	Sensitivity (%)	Specificity (%)	PPV (%)	NPV (%)	Youden’s Index
In-sample fitting	0.930	79.4	95.7	96.4	75.9	0.751
Leave-one-out cross-validation	0.857	76.5	82.6	86.7	70.4	0.591
External independent cohort validation	0.904	65.2	92.9	93.8	61.9	0.581

The optimal threshold was identified via LOOCV in the derivation cohort and applied to the external cohort. The external cohort consisted of 37 DOC inpatients from the same hospital recruited in a later period, with consistent inclusion/exclusion criteria, qEEG recording, CRS-R assessment and 6-month GOS outcome ascertainment; AUC, area under the curve; PPV, positive predictive value; NPV, negative predictive value.

## Data Availability

The datasets supporting the findings of this study are not publicly available owing to participant privacy and confidentiality restrictions. Qualified researchers with legitimate research requests may contact the corresponding author to apply for authorized access to the study data.

## Supplementary Materials

The following supporting information can be downloaded at: https://www.mdpi.com/article/10.3390/brainsci16101138/s1, Figure S1: Schematic illustration of the bedside qEEG recording setup.
