# Supplementary material for: Auditory Stimulation-Evoked Frontoparietal Theta Asymmetry for Subtype Classification and Long-Term Prognosis Prediction in Disorders of Consciousness
Source: Brain Sci. 2026 Oct 21;16(10):1138. doi: 10.3390/brainsci16101138 (PMC13702166; doi:10.3390/brainsci16101138)
Supplement: Supplementary file 1 [file brainsci-16-01138-s001.zip › brainsci-4574138-supplementary.pdf]

Supplementary Materials

Supplementary Figure S1. Schematic illustration of the bedside qEEG recording setup.

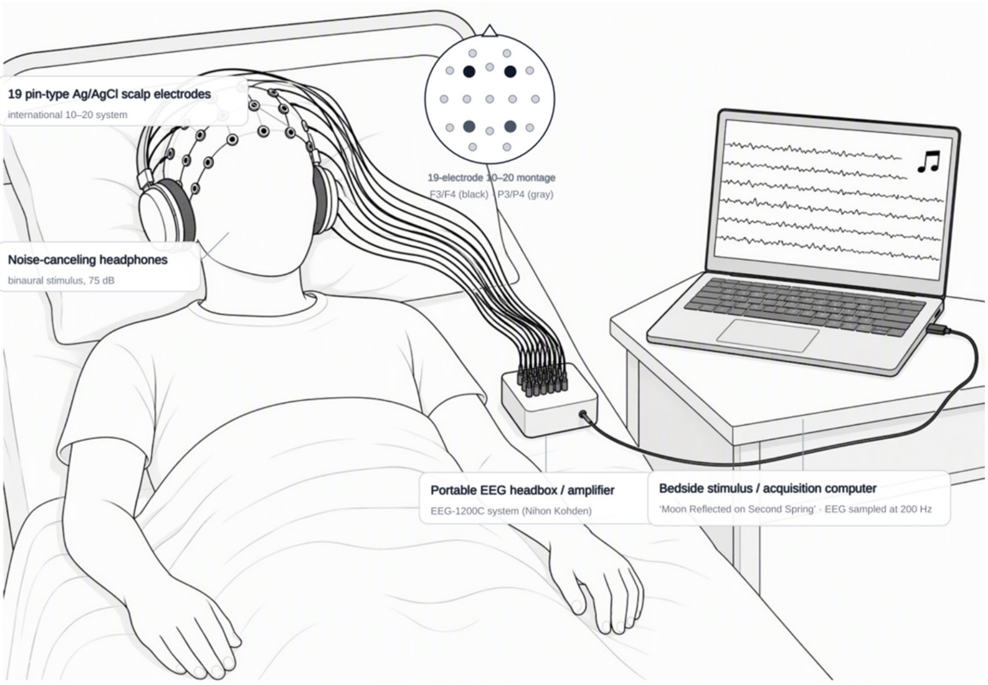

Continuous EEG was recorded using 19 pin-type Ag/AgCl scalp electrodes positioned according to the international 10–20 system. Auditory stimulation (a clipped segment of Moon Reflected on the Second Spring) was delivered binaurally at a calibrated constant volume of 75 dB through noise-canceling headphones controlled by the bedside computer. Resting-state EEG was recorded for at least 8 min before stimulation, and EEG was acquired continuously throughout music presentation. The inset shows the 19-electrode montage, with the frontal (F3/F4) and parietal (P3/P4) electrode pairs used for the asymmetry analyses highlighted.
